# Up2U: designing and validating a new evidence-based programme for perpetrators of domestic abuse who want to change

**DOI:** 10.3389/fpsyg.2025.1676490

**Published:** 2025-11-28

**Authors:** Terri Cole, Louise Oliver, Orlanda Harvey, Jane Healy, Anisha Sperryn, Arianna Barbin

**Affiliations:** Bournemouth University, Poole, United Kingdom

**Keywords:** domestic violence, domestic abuse, perpetrators, interventions, programme evaluation, intimate partner violence and abuse

## Abstract

**Introduction:**

Domestic abuse is a pervasive issue rooted in patterns of power and control, contributing to a significant number of high-harm offenses both in the United Kingdom and internationally. While programmes have been aiming to disrupt abusive cycles through the understanding and recognition of harm, there is widespread disagreement on what effective interventions should look like. This study addresses this gap.

**Methods:**

A mixed-methods, multi-phase approach was adopted to evaluate the Up2U programme in Dorset, identify current challenges, and offer recommendations for future design based on evidence and best practice. A literature review, a workshop with key stakeholders, interviews, questionnaires, and a focus group with programme providers were conducted.

**Results:**

Crucial needs were identified, including the implementation of tailored interventions, hiring skilled facilitators, supporting victims, promoting perpetrator accountability, refining the programme structure, investing in multi-agency collaborations, evaluation, and engagement/retention.

**Conclusion:**

Recommendations were drafted aligned with Home Office standards for domestic abuse perpetrator intervention. Designing flexible, evidence-based programmes that center on victim safety while holding perpetrators accountable was a crucial element of the study. Well-trained, supported facilitators and accessible, engaging content are essential for meaningful participation. Ongoing monitoring and impact evaluation to track behavioral changes are proposed.

## Introduction

Domestic abuse (DA) is a widespread issue grounded in patterns of power and control (Davies, 2018), accounting for a considerable volume of high-harm offenses^1^ nationally and internationally (Home Office, 2022). Legislatively, DA encompasses a range of abusive behaviors—including but not limited to physical, sexual, emotional, psychological, or economic abuse, as well as coercive and controlling behaviors—occurring between individuals who are personally connected and aged 16 years or older (Domestic Abuse Act, 2021). DA occurs both as single isolated incidents as well as part of persistent abusive conduct [Crown Prosecution Service (CPS), 2022] and can affect people irrespective of gender, age, disability, race, religion, sexual orientation, or gender identity, meaning anyone can become a victim (Boyd et al., 2023; Ross, 2012). Despite this, research shows that women appear disproportionately more likely to be affected, with data from 2022 to 2023 indicating that 73.5% of DA victim/survivors were women, and most perpetrators were men (Office for National Statistics, 2024; Women's Aid, 2019). Proportionally, it is also documented that the scale of DA is substantial, with over 9.9 million people reportedly experiencing it at least once across their lives (Crime Survey for England and Wales; CSEW, 2024), and 2.3 million (4.8% of the population) being affected in the past year (Office for National Statistics, 2023). Although under-reported—less than 24% of victims contact the police following abuse—it is estimated that DA is present in at least 16.2% of recorded offenses in England and Wales (Office for National Statistics, 2023; Refuge, 2022).

Variation across crimes occurring within DA contexts has also been reported. Violence with injury, for example, was recorded in over 60% of DA police reports to the police (Crivatu et al., 2025). In some cases, the violence escalated lethally, with 66 of 108 domestic homicides (61.1%) recorded between March 2023 and 2024 being perpetrated by partners and ex-partners of the victims (Office for National Statistics, 2024). Concerningly, in over 90% of police DA-flagged cases, the suspect was not charged, and the odds of this outcome were 57.9% lower if the victim was also raped or sexually assaulted during the incident (Barbin et al., 2025). In this regard, Barbin et al. (2025) highlighted that the most common DA outcomes were either the victim not supporting prosecution^2^ (*N* = 87,813, 50.2%) or the law not pursuing the allegations^3^ (*N* = 61,028, 34.8%).

The consequences of DA are well-documented in the literature, with authors reporting the long-lasting and severe effects on survivors' physical, economic, and psychological wellbeing (Stubbs and Szoeke, 2022; White et al., 2024). This effect extends beyond the immediate victim/survivor, with children (directly and indirectly witnessing DA) facing adverse emotional, behavioral, cognitive, and social experiences (Evans et al., 2008; Evans and Hotten, 2022; Walker-Descartes et al., 2021). As a result of these factors, legislative changes have been implemented to recognize children as DA victims in their own right (Domestic Abuse Act, 2021).

During the COVID-19 pandemic, DA incidents surged exponentially following the implementation of national lockdown measures, financial strain, challenges in reporting crime, and distress among the population (Clemente-Suárez et al., 2021; Spence et al., 2022). Although DA reports have since plateaued, more study is needed to improve DA prevention, training, and prosecution (Office for National Statistics, 2024). First, programmes addressing DA incidents are grounded in the idea that a small, high-risk population is responsible for a disproportionate amount of serious offending (Robinson and Clancy, 2021), often characterized by a documented history of violent behavior and recidivism (Klein and Tobin, 2008; Ministry of Justice, 2024; Sechrist and Weil, 2018). In line with these findings, efforts have been made to prioritize interventions for repeat and violent DA offenders, as these are critical to reducing further DA offending and promoting victims' safety; a position that was legislatively reinforced by the Istanbul Convention (Council of Europe, 2011).

Programmes have been aiming to disrupt abusive cycles by promoting suspects' accountability and supporting behavioral change through the understanding and recognition of harm (Bolden, 2010). Some of these programmes target intimate partner violence and abuse, like the Duluth Model, focusing on patriarchal structures influencing perspectives and behaviors as triggers for DA normalization (Babcock et al., 2016; Cannon et al., 2016). Despite being one of the earliest programmes to target DA, the Duluth Model has been criticized in more contemporary discourses as it neglects less “stereotypical” forms of DA (like female-to-male DA, same-sex offending, etc.), alongside not being victim-centered, disregarding potential risk factors and bypassing treatment needs (Birkley and Eckhardt, 2015; Bohall et al., 2016; Ehrensaft, 2008; Pender, 2012).

Cognitive–behavioral therapy (CBT) models have been developed as an alternative to address maladaptive thoughts and behaviors to improve healthy skills development and promote intrinsic change (Dutton and Corvo, 2007; Murphy et al., 2020). A meta-analysis of programmes designed to reduce recidivism among offenders found that CBT, on which the Duluth model is also based, did not produce convincing results related to reduced recidivism (Gannon et al., 2019). Early empirical studies on DA perpetrator programmes found little evidence of CBT effectiveness, with programmes showing no differences between control and intervention groups, only slightly decreasing recidivism upon completion, or yielding mixed results (Davis et al., 2000; Rosenfeld, 1992). A rationale for CBT ineffectiveness in reducing future engagement with crime might be due to the method underpinnings that require individuals to be promoters of their own behavioral change (Hofmann et al., 2012). Within DA contexts, this requirement is further exacerbated, with evidence showing that CBT is inconclusive in eliciting change in the thinking patterns and behaviors of individuals abusing their partners (Smedslund et al., 2011).

As CBT programmes became increasingly mandatory through arrest and prosecution policies (Wilson et al., 2021), the research focus has been moved to measuring interventions' effectiveness—as opposed to changes in recidivism rates^4^ (Saunders, 2008). The findings prompted a need to improve programmes, not only through effective design and delivery, but also more appropriate evaluations targeting differences across what makes programmes effective and which factors can deter future recidivism (Andrews and Dowden, 2005; Bowen and Gilchrist, 2004; Esbensen et al., 2011).

In line with this, the current study aimed to evaluate and identify challenges in a newly developed DA programme, called Up2U—Creating Healthy Relationships^5^, and incorporate identified best practices and tailored recommendations to improve client outcomes. Up2U is a programme for males and females aged 16 and older and is for both heterosexual and same-sex relationships. Participants must admit that they use abusive behaviors in their relationships and want to change them (BCP Council, no date). Up2U draws on a range of different tools and techniques to support change thinking in its programme and takes a “neutral stance” in the debate between models, recognizing that DA is often about exerting power and control over the abused partner, and other instances must consider additional risk factors on an incident-to-incident basis. Up2U's neutral stance also adds to previous models by looking at DA dynamics, regardless of demographic characteristics of perpetrators and victims (e.g., whether they are men or women, or whether they are in same-sex vs. opposite-sex relationships).

## Materials and methods

A multi-phase, mixed-methods design was implemented, with the study drafted as a participatory and reflective enquiry of the Up2U programme. The methodology integrated practitioners, stakeholders, and service user perspectives on the programme, aiming to gather a comprehensive, evidence-based, and contextually informed understanding of the programme's effectiveness and hindrances in deterring further abusive behaviors. To achieve the study's aim, five phases were designed. Ethical approval was granted for each phase by [redacted for review] Ethics Panel on the 26th of September 2024 [Ref id: 58713].

Phase 1 consisted of a literature review to investigate effective and ineffective practices around perpetrator programmes. In line with Cooperrider et al. (2007), Phase 2 involved an in-person Appreciative Inquiry (App Inq) through a facilitated workshop with 25 adult professionals from the criminal justice, health, victim services, and social care sectors who work closely with DA victims/survivors and perpetrators. Participants were recruited via the research team and funders contacting key gatekeepers within their professional networks and asking them to contribute. The workshop participants received a brief introduction to App Inq and the session's context. Being familiar with Up2U was not a requirement for participation, although those already working in the programme were grouped together (n=5). The remaining participants (*n* = 20) were split into four additional randomized groups, each led by a facilitator. Participants engaged in structured group discussions on good practice (labeled as the *discover phase*), brainstormed ideas on how to improve the support available to victims (*dream phase*), and made some suggestions for more effective practice (*design phase*) using Ketso© boards^6^. The second phase focused on prioritizing strength-based reflections and multi-agency collaborative ideation.

Phase 3 consisted of semi-structured interviews with approaching eleven additional individuals linked to (i.e., victims/survivors) or participating in the Up2U programme. At this time, UP2U only had male clients who used abusive behaviors. Three of these agreed to take part (27.3% participation rate): two DA perpetrators (male) and one victim/survivor (female). The interviews, conducted online via Microsoft Teams, explored the lived experiences of Up2U programme users (or their partners in the case of the victim/survivor) to identify areas for improvement. Participants were allowed to keep their cameras off, and audio recordings were retained. Participants were provided with additional support/debrief details at the end of the interview due to the sensitivity of the topic discussed. Due to the small sample of people recruited during Phase 3, participants' experiences of Up2U were collected (Phase 4) via a secondary data analysis of 24 anonymized service user questionnaires shared by Up2U^7^. Responses were analyzed based on both areas of the programme that clients (men who used abusive behaviors) found helpful, as well as aspects of Up2U that they wanted to see improved. Finally, Phase 5 entailed an in-person focus group with programme practitioners to capture their insights and critical reflections on delivery, grounded in prior knowledge of the Up2U model (Ford, 2019). Data from all five phases were triangulated to provide a nuanced evaluation of the programme processes, outcomes, and potential.

### Procedure and data analysis

Thematic analysis was undertaken for each phase using an adaption of the thematic networks process. This process seeks to identify one overarching global theme (a superordinate theme which encompasses the data as a whole) from related organizing themes, which organize basic themes into clusters of similar issues; and basic themes derived directly from the data (Attride-Stirling, 2001). The data were organized in *basic themes* (derived directly from the text), before creating *organizing themes* across phases, to generate an overarching *global theme* capable of encapsulating the entire dataset. Phases 1 and 2 allowed for the creation of *basic themes* and preliminary *organizing themes*, generated from both the literature and discussions with professionals. Phases 3 and 4 allowed for qualitative and quantitative themes' refinement through semi-structured interviews and the Up2U survey, making space for additional themes to be included. Finally, in Phase 5, focus groups were transcribed and coded to incorporate new data on the expanding thematic structure. Once each phase was completed, all themes were re-analyzed collectively to develop the overarching global theme. Participants' data were anonymized, and pseudonyms were used across all phases to protect anonymity and confidentiality.

## Results

**Figure 1:**
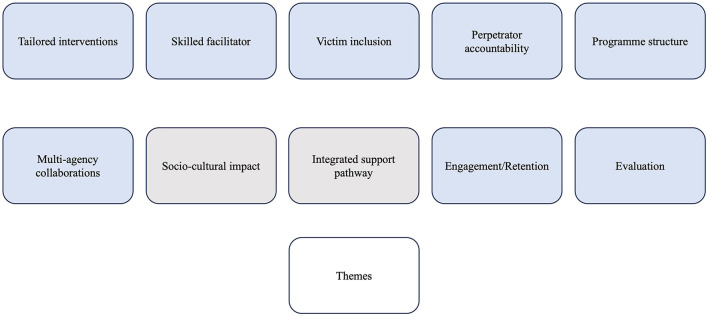
Study themes identified across the five phases. Themes reported in gray originated from the data and those from the literature review are in blue.

The study progressed iteratively, with each phase designed to explore emerging themes in greater depth across different stakeholder perspectives. This layered approach enabled validation, contrast, and enrichment of themes across the literature, professional practice, and lived experience. Ten overarching themes emerged: eight from the literature and two from the research (Figure 1). Their recurrence and development across each phase are outlined in Table 1.

**Table 1:** Themes development across phases.

**Theme**	**Phase 1 (lit review)**	**Phase 2 (workshop)**	**Phase 3 (interviews)**	**Phase 4 (survey)**	**Phase 5 (focus group)**
1. Tailored interventions	Introduced the need for personalized approaches based on typologies, risk, culture, and learning needs.	Need for a systemic approach to support and cross-agency collaborations.	Importance of adapting in real time to meet individuals' needs.	Identified failures when programmes did not account for the victim's context.	Described challenges in connecting generic material to personal experience.
2. Skilled facilitator	Emphasized the role of well-trained facilitators in effectiveness.	Value of supervision, training and career development, networking, and counseling.	Detailed the benefits of personalized interventions and praised the facilitator's understanding.	Facilitators were labeled as non-judgmental and held in very high regard.	Acknowledged challenges in recruiting and retaining skilled facilitators.
3. Victim inclusion	Argued for the integration of victims/survivors' feedback and safety planning.	Emphasized how the programme could be improved by supporting the victims.	Discussed from both the perspective of perpetrators and victims.	Emphasized how the programme could be improved by also engaging with victims.	Acknowledged that victims' voices and feedback could enhance accountability and progress.
4. Perpetrator accountability	Highlighted the importance of challenging denial/minimization.	Some perpetrators show minimization of their actions; the programme seen as a tool to address this.	Explored the perpetrator's reflexivity.	Victims questioned whether real accountability is achieved.	Language-based challenges raised due to the stigma attached to the word perpetrator
5. Programme structure	Reviewed effective programme models and theoretical grounding.		Interviewees noted diverging victims/perpetrators' perspectives.	Clear aims, but room for relevant and realistic content improvement, especially around gender-bias.	Focus on how to improve the programme structure and remove outdated material.
6. Multi-agency collaboration	Advocated joined-up responses across services.	Better information streamlining is required for risk assessment and support.	Explored poor communication and disjointed risk management.	Noted the need for support services to be clued up on programme content.	Perpetrators noted confusion around referral and support pathways.
7. Evaluation	Discussed the limits of recidivism data and the value of mixed measures.	Noted that evaluation was needed but resource-intensive	Stressed need for ongoing feedback and learning loops.	Wanted programmes judged on lived change, not completion.	Supported follow-up, but unclear what “success” meant for them.
8. Engagement and retention	Noted need for adequate investment in skilled provision.		Raised concerns over caseloads and emotional toll.		Recognized pressure on facilitators and inconsistency in support.
9. Socio-cultural impact		Stereotypes and societal norms are impacting the behaviors of those in relationships.			Practitioners raised actively working to limit the influence of normalized gender bias.
10. Integrated support		Recognized that support should be holistic, and all aspects of support needed funding		Referred individuals reported wishing more people were aware of the programme.	Frustrations around the lack of resources to extend accessibility to all DA perpetrators were raised.

### Theme one: tailored interventions

Evidence from the literature highlights the importance of tailoring interventions to the diverse profiles and needs of DA perpetrators (Butters et al., 2021), as their behaviors, underlying motivations, and psychological triggers can differ significantly (Haselschwerdt et al., 2019; Murphy and Meis, 2008). Overlooking tailored risk assessments and contextual influencing factors can risk missing critical drivers of abuse, leading to reduced programme effectiveness (Capaldi and Kim, 2007; Johnson, 2008). When tailoring interventions to DA perpetrators, like for the Duluth model, evidence from the literature suggests that integrating relatable content leads to better and more meaningful engagement from referred participants (Contrino et al., 2007).

Reflecting on this consideration, one of the perpetrators interviewed (*Phase 3*) recognized the value and importance of having participated in tailored sessions, speaking of how understood he felt by his facilitator:

“*He's very good […] he keeps it real. I can't stand it when you're sat there and preached to by somebody's, and you know they're just reciting a corporate manual or a book that they learnt whilst they were at university. It's about bringing it to life so people can relate” (Rob)*.

In this regard, most survey respondents (*Phase 4*) agreed that the methods used by Up2U facilitators met their individual needs and were adequately tailored to respond to their needs. Evidence from the literature also underscores the importance of making programmes more inclusive to reflect diverse backgrounds and individual challenges relating to gender identity, age, disability, trauma, or substance misuse, as doing so reduces participants' alienation and withdrawal (Bates et al., 2017). An example of adaptation is reported in the study by Banting et al. (2018), who modified Solution-Focused Brief Therapy to support a male DA perpetrator with a learning difficulty. The adjustments included the use of written prompts, bullet points, visual emotional charts, and role-play activities, allowing the participant to engage more directly, actively, and reflectively with the programme. Consistently, focus groups with practitioners (*Phase 5*) reiterated how the programme structure, activities, and exercises are adapted for each client, leveraging on topics that are of interest to them, as well as analogies they understand and relate to:

“*No two people get the same version of the programme because of it being [tailored] to that individual; adapted accordingly” (Tom)*.

Additional concerns raised in the literature include the presence of deficits in emotional processing, such as alexithymia, among DA perpetrators (McGinn et al., 2020; Strickland et al., 2017). Failing to adjust programmes for emotional dysregulation, cognitive distortions, and low self-esteem has been shown to compromise DA programmes' effectiveness, particularly for referred individuals who struggle with communication and frustration tolerance (Engin et al., 2010; Mattila et al., 2010; Ogrodniczuk et al., 2011). Tailored support is also lacking for marginalized groups (Turhan, 2020), with cultural considerations often overlooked, and only a few programmes accommodate LGBTQ+ needs (Morgan et al., 2019). Designing bespoke, culturally sensitive interventions improved engagement (Hancock and Siu, 2009), decreased stigma among minority men (Carbajosa et al., 2017; Kim, 2010), and improved accessibility (Turhan, 2020). Practitioners (*Phase 5*) also reported that there is a requirement to unpick individual perpetrators' behaviors and help them to reframe their actions, exploring broader concerns and constraints (e.g., financial, geographical, familial).

The workshop conducted in *Phase 2* also highlighted the importance of person-centered and trauma-informed approaches, underpinned by the belief that people *can* change and should be supported to do so. Practitioners emphasized the importance of adequate supervision, both managerial and peer, to safeguard wellbeing and prevent bias. Crucially, practitioners believed interventions should be embedded within a broader system of sustained support, including services for victims/survivors and children, especially in instances where the relationships continue during programme attendance. One-off interventions were deemed insufficient, with practitioners recommending long-term and group involvement, as well as mentorship, and post-completion support. A need for early intervention, restorative justice, and tailored services for young people was also raised, alongside the identification of systemic barriers (e.g., inconsistent data sharing, resource gaps, and fragmented referral processes) that can hinder the effectiveness of local responses.

### Theme two: skilled facilitator

Effective delivery of DA perpetrator programmes relies heavily on the skill and preparedness of facilitators (Pallatino et al., 2019). Despite this, in the literature, many facilitators are reportedly underprepared and unsupported when dealing with victim/survivors and perpetrators. According to HM Inspectorate of Probation (2018) and Renehan (2021), several practical constraints, such as training gaps, workload, and emotional strain, can hinder facilitator effectiveness when navigating the programme and negatively affect their wellbeing. Exposure to traumatic narratives and details can lead to both vicarious and secondary trauma, especially in facilitators with personal experiences of abuse (Cluley and Marston, 2018; Goldhill, 2019). These issues highlight the complex emotional and professional demands placed on facilitators, underscoring the need for sustained support structures. Addressing these concerns is crucial, as poorly trained facilitators have also been shown to mishandle sensitive discussions, fail to challenge harmful beliefs, or inadvertently perpetuate victim-blaming (Hughes, 2017). The finding is especially concerning in group settings, where McGinn et al. (2020) found participants occasionally experienced (or witnessed) minimizations of the abusive behaviors recounted. Pender (2012) found that basic training rarely prepares facilitators to effectively manage these dynamics, which may result in collusion among participants or failure to confront abusive attitudes. Challenges associated with recruiting and retaining highly skilled facilitators were also raised in the focus groups (*Phase 5*). To address this, facilitators need comprehensive training, supervision, and emotional support (Evans and Hotten, 2022; Taylor et al., 2020). Programmes like the DARE toolkit (DARE - Hampton Trust, Breaking the cycle of abuse, n.d.) and RESPECT appear to offer practical tools for identifying risk and engaging perpetrators. The ADVANCE-D Programme | ADVANCE-D (2024) also provides training to monitor co-occurring issues such as substance misuse, which is essential for tailoring interventions to complex DA cases. Equally important is critically reflective practice, which supports facilitators in maintaining emotional safety in both group and one-on-one settings. During *Phase 5*, supervision was also reported as vital to maintain the wellbeing and effectiveness of the support provided. Critically, reflective practice reportedly helped the facilitators to manage their expectations and self-reflect on their work with suspects, particularly in circumstances where they were at risk of being manipulated (e.g., during interviews). The literature also suggests that reflexivity can help balance empathy with accountability, challenging harmful behaviors while supporting the individual's capacity to change, making it a crucial step of facilitators' toolkits (Hamel, 2012; Hughes, 2017; Silvergleid and Mankowski, 2006). Practitioners (*Phase 2*) also mentioned that greater emphasis should be placed on training and career development around key skills, behaviors, and techniques. Networking, counseling, and wellbeing support for staff, especially if working in contexts of safeguarding, were also pinpointed.

In terms of Up2U clients, both interviewed perpetrators (*Phase 3*) were very complimentary of the facilitators' skills. When pushed about specifically what they felt was useful, they reflected on how the facilitators' non-judgmental attitude made them more likely to consider personal behavioral change:

“*He wasn't giving his opinion, but helping me pick and understand [my thoughts]. I could have acted differently, and we would talk that through it […] he doesn't patronize me, summarize me. He'll plant the seed and let me go away and do it” (Rob)*.

“*You can sit there and speak to him, not feel judged… he's very understanding about it” (Ben)*

These perspectives were also supported in the survey (*Phase 4*), with facilitators being labeled as professional, respectful, and engaged, staying in regular contact with them throughout the programme. Thus, Up2U facilitators were held in very high regard by their clients. It was clear from the subsequent phase that facilitators were highly skilled and actively contributed with their expertise and experience to the programme, e.g., one facilitator shared how they used a shared taste in music to build rapport and help engage the client. They were reportedly able to adapt the programme to meet individual learning needs, showing awareness of the impact of certain activities on their clients' beliefs and engagement.

### Theme three: victim/survivor inclusion

Victim/survivor perspectives are essential to the effectiveness of perpetrator programmes. Influenced by increased feminist advocacy, policy has gradually shifted from passive responses to proactive strategies that prioritize safety and support in DA contexts (Tapley, 2010). While programmes aim to change perpetrator behavior, they must also ensure that victim safety remains central, as survivors consistently emphasize the importance of long-term support and safety (McGinn et al., 2021). Earlier interventions, such as the Integrated Domestic Abuse Programme (IDAP), focused on behavior change but often failed to integrate victim safety as a core concern (Eadie and Knight, 2002) and lacked adequate support structures for women (Bilby and Hatcher, 2004). Bullock's (2014) evaluation of Women's Safety Work revealed that such services frequently fell short of their stated aims, offering limited contact and engagement with victims/survivors. In response, more recent programmes like DRIVE, RESPECT, and the Domestic Violence Intervention Project have adopted more holistic models that include continuous support for victims, such as regular risk assessments, monitoring, and comprehensive wraparound services (The Drive Project – The Drive Partnership, n.d.; DRIVE, n.d.). Notably, the DRIVE model has reported moderate to significant risk reductions in 82% of cases. Despite the progress achieved, many gaps remain. Bates et al. (2019) found that in 72% of UK organizations delivering perpetrator programmes, facilitators had no contact with victims/survivors, although some organizations offered auxiliary support, including peer groups, legal assistance, housing help, and mental health services. While growing awareness was documented, inconsistencies and the need for more structured, victim-focused provision were also highlighted. A key concern is that presenting interventions as rehabilitative may give victims false reassurance, especially if genuine behavioral change has not yet occurred (Holtzworth-Munroe et al., 1995; McGinn et al., 2020). Victim inclusion extended to survivors feeling unable to corroborate perpetrators' statements (*Phase 3*), unsure of whether the programme can lead to effective change:

“*I would love to know what the programme entailed. I've got no idea what they discuss or what they follow. He'll say things […] but whether that's the truth or not […] he doesn't tell the truth a lot of the time”*.

One survey respondent (*Phase 4*) noted that Up2U could be improved by giving the chance to both perpetrators and victim/survivors (e.g., ex-partners) to complete the course. Interestingly, it was the intention that the Up2U programme should have a mirrored programme for victims/survivors, but resources prevented this from occurring.

Incorporating victim-informed approaches through ongoing safety planning, individualized support, and active monitoring can reduce the risk of repeat harm and ensure more robust protection (Hester and Westmarland, 2006). When implemented safely, feedback mechanisms from victim/survivors can offer valuable insights into the effectiveness of interventions and help improve overall service delivery [Crown Prosecution Service (CPS), 2022; Kropp, 2009]. Victim/survivor inclusion also involved making perpetrators reflect on the impact of their actions on survivors. For instance, during one of the interviews (*Phase 3*), one participant was encouraged to take the victim's point of view by actively reflecting on the impact of the abuse perpetrated and comparing how the victim might have felt with his perceptions of the crime. Having knowledge of the case details also enabled facilitators to corroborate or challenge the participant's statements where required, as well as allowed them to promote accountability.

### Theme four: perpetrator accountability

Promoting accountability is essential to the success of perpetrator programmes, particularly as many individuals appear to minimize or deny their abusive behavior (Hamberger and Guse, 2002; Wilczynski et al., 2012). Hughes (2017) found that facilitators criticized earlier programme models for failing to challenge abusive attitudes directly, warning that vague goals can allow perpetrators to avoid responsibility and bypass critical behavior change. In contrast, RESPECT-accredited interventions, such as The Change Project, focus on fostering self-awareness and personal responsibility, with reported reductions in women's injuries from 61% to just 2% following programme completion (Respect, 2022). Early intervention models, such as Make a Change, developed in partnership with Women's Aid, aim to disrupt harmful behavior before it escalates into criminality (Make a Change, n.d.). Similarly, the DRIVE programme offers intensive support to confront and shift controlling, violent, or gendered beliefs, replacing them with non-abusive coping mechanisms (DRIVE, n.d.). Crucially, effective programmes ensure that perpetrators do not externalize blame, as factors such as stress, alcohol, or a partner's actions must never be seen as justifications for abuse. In this regard, one of the perpetrators elaborated (*Phase 3*) on how the programme forced them to reflect on past behavior to integrate long-term change:

“*[They were] trying to get me to reflect and be more aware of my own feelings and what my response is because I can [be] calm, but I'm […] sort of explosive. I'll get very irritated, and I'll say the wrong thing and then, you know, I may regret it later” (Ben)*.

Sustained behavior change relies on a structured approach that reinforces personal accountability through professional challenge, consistent support, and coordinated community oversight. Conversely, from victims' perspectives, this might also mean reflecting on the lack of perpetrator accountability despite joining the programme:

“*{it is] almost giving him reasons for what he has done, [saying] there is a reason why he's done these things […]he can see what he's done, but I wouldn't say he acknowledges what it is. I can see his circle […] and when he goes into different patterns. But I don't think he has any awareness of that. He still doesn't take any responsibility for what happened”*.

Deflections of responsibility and accountability were also reported in the survey (*Phase 4*). It was therefore suggested in the following phase (5) that the programme should, as a core theme, include perpetrator accountability. The finding implies encouraging those who use abusive behaviors to think carefully about the impact these behaviors have on others, supporting them to take responsibility for their current circumstances (e.g., working toward achievable goals, challenging harmful or abusive behaviors, and eliciting holistic positive change):

“*Helping them to understand that we can [help them] make incremental steps toward what they want […] creating [a] bigger safety net in terms of quality of life and then they [can] feel more ownership from it and [be] more grounded in their responsibilities and awareness” (Tom)*.

### Theme five: programme structure

Dixon et al. (2012) emphasize that perpetrator programmes should address criminogenic risks from a therapeutic as well as from a purely educational perspective. Bates et al. (2017), through a review of 21 UK-based organizations, found that most programmes aim to improve emotional regulation, communication, coping, and life skills (100%), and often include anger management, impulse control (95.2%), and awareness of the impact on victims and children (90.5%). Programmes also address power and control dynamics (81%), gender roles (76.2%), and seek to challenge harmful, pro-violent beliefs (71.4%). Common techniques include role-play (95.2%), meditation (76.2%), assertiveness training (66.7%), and journaling (61.9%). While these features appear constructive, concerns remain about effective programme delivery. Hughes (2017) reported that Building Better Relationships (BBR) was overly rigid, with up to 20 pages of content per session, leaving little space for client engagement. Facilitators found the material complex and challenging to deliver, and participants struggled to retain the key concepts taught. McGinn et al. (2020) similarly noted that inaccessible or overly formal language can reduce engagement and motivation. Good practice involves more relatable, practical tools. Hughes (2017) found that participants remembered using the “Helicopter View” to gain perspective, recognize triggers, and manage reactions. Another widely valued strategy is the “Time Out” technique, which encourages participants to step away from escalating situations. Wistow et al. (2017) and Debbonaire et al. (2004) found this approach helped reduce abusive incidents and improved victims' sense of physical safety.

In terms of Up2U, participants highlighted specific parts they found useful (*Phase 3*); in particular, the Up2U “color technique” to check themselves and monitor their responses:”*having a breather”(Ben)* to think before acting and brainstorming different scenarios and how they could be improved. Rob also raised the importance of self-awareness and how frustrations and tensions could build up before incidents and mentioned how group sessions did not work for him:

“*Signposting of the sessions [is] really good, so I know what I'm going to be thinking about for the week before I'm coming in prepared […] I certainly wanted to go face-to-face [but] talking to somebody about these things [in] teams it didn't work for me. It [did not feel] as [real] counseling” (Rob)*.

While the programme's clients reported well-documented aims and sessions (*Phase 4*), from a victim/survivor perspective, more conversations about the topics discussed in the programme could have taken place. Sarah stressed (*Phase 3*) how perpetrators should work on developing awareness of the cycle of abuse and on becoming acquainted with what counts as abuse (including emotional and sexual), as well as an overview of how such behaviors can impact children, having and maintaining healthy boundaries, and taking other people's perspectives (understanding how the other person may feel).

Focus group participants (*Phase 5*) emphasized the need to refresh the Programme content while maintaining a strong focus on one-to-one sessions, which were seen as essential for building trust and tailoring support. Activities and tools such as “sweep to keep^8^“, “press pause,” and the transactional analysis-based “colors” activity were praised for enhancing engagement and promoting emotional awareness as one facilitator reflected on how the colors were a useful tool for asking questions about behavior change, for example: “*when you are feeling blue, that can be like resentment… and then what color are you?*” (Marina). Participants also found discussions on stereotypes and gender roles to be valuable, although some materials were outdated. Suggestions for improvement included clearer language, ensuring tools used, such as a metaphor, worked for the client, and incorporating deeper content on sexual coercion, stalking, cultural influences, and substance misuse. A greater emphasis on respect, compromise, and media portrayals of relationships was recommended, alongside more comprehensive training to equip facilitators in addressing manipulative and coercive behaviors.

### Theme six: multi-agency collaboration

Effective perpetrator interventions require more than programme completion alone, as behavior change is not guaranteed, and risks may escalate if cases are prematurely de-escalated (Verney, 2022). Given the heterogeneity among those who cause harm (Haselschwerdt et al., 2019), a multidimensional and collaborative response is reported as essential (Dutton and Golant, 2008). Historically, victim services and perpetrator programmes operated in isolation, neglecting their interdependence (Clarke and Wydall, 2013). However, multi-agency collaboration is increasingly recognized as a vital tool to improve information sharing, co-location, and multidisciplinary teams, enhancing coordinated responses and accountability (Cleaver et al., 2019). The Domestic Abuse Intervention Project (Pence and Paymar, 2003), the DRIVE programme, and RESPECT-accredited interventions exemplify integrated models that engage agencies including police, probation, health services, and victim support to manage risk and monitor change. Programmes such as BBR also use Multi-Agency Public Protection Arrangements (MAPPA) to strengthen oversight. These coordinated approaches help prevent programmes from being misused by perpetrators to further manipulate victims, ensuring a more comprehensive and sustained response to DA.

Not surprisingly, the need for effective multi-agency collaboration was highlighted in *Phase 2*. Participants shared how in “an ideal world,” information sharing would be clear and compelling, processes would be streamlined, risk assessments would be joined up, shared and updated, and there would be wrap-around support for all those who are impacted. While multi-agency collaboration could help to clarify the narratives provided by participants, the need for timely referral and multi-agency awareness of programmes is often overlooked (*Phase 3*): “*I was appalled by the fact that the social worker had no understanding of what it actually was, that she was putting me forward for. I just couldn't believe it*” (Rob).

In *Phase 5*, it was recognized that it was important for facilitators to signpost participants to relevant support services and to provide additional help where possible. It was also seen that there needed to be a clear link between the provider and the services that support victim/survivors. Finally, those initiating the referral should clearly explain the programme to the client, including the expectations and accountability for signposting to be done sensitively to avoid discouraging engagement.

### Theme seven: evaluation

Assessing the effectiveness of DA perpetrator programmes remains challenging due to substantial variation in participant profiles, programme design, outcome definitions, and follow-up durations (Akoensi et al., 2013; Arias et al., 2013; Eckhardt et al., 2006; Gondolf, 2012). Traditional evaluations based solely on programme completion or recidivism rates are insufficient; instead, recent research calls for incorporating the perspectives of partners and children, as well as multi-agency reports (Vall et al., 2024). Project Mirabal exemplifies this approach, evaluating 11 programmes across multiple domains, including communication, parenting, and behavioral change. It demonstrated significant reductions in various forms of abuse and a 51% increase in women's perceived safety 12 months post-programme (Verney, 2022). Yet, as only a quarter of studies in Vall et al. (2024) review included ex-partner reports, comprehensive outcome measurement remains rare. Lilley-Walker et al. (2018) proposed a systematic model for evaluating programmes, advocating standardized definitions, detailed participant and facilitator data, mixed methods, and long-term follow-up. However, Turner et al. (2023) highlighted ongoing gaps in these areas, with limited consistency in how studies define, document, and measure success. Without more rigorous and standardized evaluation frameworks, it remains challenging to determine which interventions are genuinely effective and under what conditions. Measurement challenges are also associated with the widespread use of self-reported measures to gauge whether positive change has been elicited (*Phase 4*).

In *Phase 5*, a range of sources, including policing reoffending rates and social worker feedback, were scrutinized. Conditional measures of success for Up2U were also assessed through engagement levels and stability while on the programme, as well as programme-related cost savings. Despite the overall satisfaction with the evaluation tools available, the lack of feedback from victim/survivors was raised as a missing piece for more effective intervention outcomes.

### Theme eight: engagement and retention

The most documented challenge to the effectiveness of DA Perpetrator programmes is the high attrition rates, with dropout figures ranging from 0% to 64% (Vall et al., 2024). Attrition has been shown to undermine programme impact, as one early study found completion is linked to reductions in DA by up to 64% (Gondolf and Jones, 2001), while non-completion can predict future harm (Lauch et al., 2017; Lila et al., 2019). Factors influencing dropout include individual characteristics such as age, education, prior convictions, and mental health (Cunha et al., 2023), with many dropout indicators being predictors of recidivism (Cattaneo and Goodman, 2005). Motivation is another key factor; court-mandated participants tend to be less engaged than voluntary attendees, whose intrinsic motivation is associated with better outcomes (McGinn et al., 2020; Parhar et al., 2008). Motivational interviewing has shown promise in enhancing commitment and reducing attrition (Pinto e Silva et al., 2023). However, even voluntary referrals face challenges, with many disengaging before the programme begins, often influenced by facilitator skills and confidence (Donovan and Griffiths, 2015). Although group programmes can support accountability and reduce denial (Simmons, 2009), they may also foster harmful dynamics or peer validation of abusive behaviors. For example, engagement is not always genuine (e.g., some individuals attend solely to influence court or child protection outcomes), resulting in superficial compliance rather than real change (McGinn et al., 2020). These findings underscore the importance of careful assessment, targeted support, and ongoing monitoring to enhance retention and ensure meaningful participation.

In *Phase 3*, referred participants highlighted how a lack of clear information and preparation can undermine early engagement with Domestic Abuse Perpetrator Programmes. Rob's experience shows that not knowing what to expect made him uncertain, suggesting that a simple information pack or pre-meeting could improve understanding and willingness to participate. Ben's initial skepticism shifted once he learned the sessions were one-to-one, helping him feel less stigmatized. His comments also show that the facilitator approach and one-to-one interventions made him feel safe enough to continue. These insights align with broader research indicating that early communication, personal relevance, and delivery style are crucial factors in enhancing engagement and reducing dropout rates. In the last *Phase* (5), tension was acknowledged between the need for participants to attend and engage with the programme and course material and the need to hold them accountable. Steps taken to encourage engagement included allowing participants the choice of whether to attend appointments and being very careful in the use of language to promote engagement and participation. It was also crucial that sessions did not feel repetitive for participants.

### Theme nine: socio-cultural impact

One of two themes developed directly from participants, rather than drawn from the literature, was the need to consider the wider societal and cultural impact of DA within programmes. In *Phase 2*, participants emphasized how gendered stereotypes and contextual social norms reinforce harmful behaviors in relationships, particularly among men (who are the target clients for Up2U). They highlighted the importance of early intervention through education in schools on healthy relationships and emotional intelligence, as well as a greater responsibility for professionals to challenge these norms. There was a strong view that current narratives around gender roles, such as men being expected to lead households and women to care for children, continue to shape behavior and limit help-seeking in DA contexts. These persistent norms were seen as contributing to the problem and in need of a significant, systemic change. In *Phase 5*, practitioners mentioned already being in the process of challenging gender norms and harmful stereotypes expressed by suspects during programme delivery, particularly around intimacy, violence, and gender roles. The discussion, although brief on the topic, indicates a growing awareness of how broader social messages can shape the normalization of abusive behaviors. The idea of challenging norms also aligns with the wider theme of the need for systemic change, as it demonstrates that even within individual sessions, these societal influences are present and must be actively unpacked. The mention of cultural and gender-specific needs further supports this, pointing to the importance of tailoring interventions to reflect the diverse ways in which social norms may impact different groups.

### Theme ten: integrated support

In *Phase 5*, focus group participants shared an ethos that support should be available to all those who need and want it. For example, a common logistical issue across regions is the fact that some only offer support and referrals if the perpetrator and victim have shared custody of a child. While this is a limitation linked to the availability of funding sources, practitioners expressed widespread frustration.

“*For me, if somebody is asking for help because they've recognized and want to be accountable for their domestic abuse, perpetrator behavior, how can we not take them on to a domestic abuse perpetrator programme. That doesn't compute with me at all” (Linda)*.

In *Phase 4*, participants not only appreciated Up2U but stressed the need for earlier intervention and shorter waiting times, echoing the practitioners' concerns about missed opportunities for change, mentioning how they wished more people had the chance to join the programme. Together, these insights strengthen the argument for more inclusive, timely, and sustained access to perpetrator programmes.

**Figure 2:**
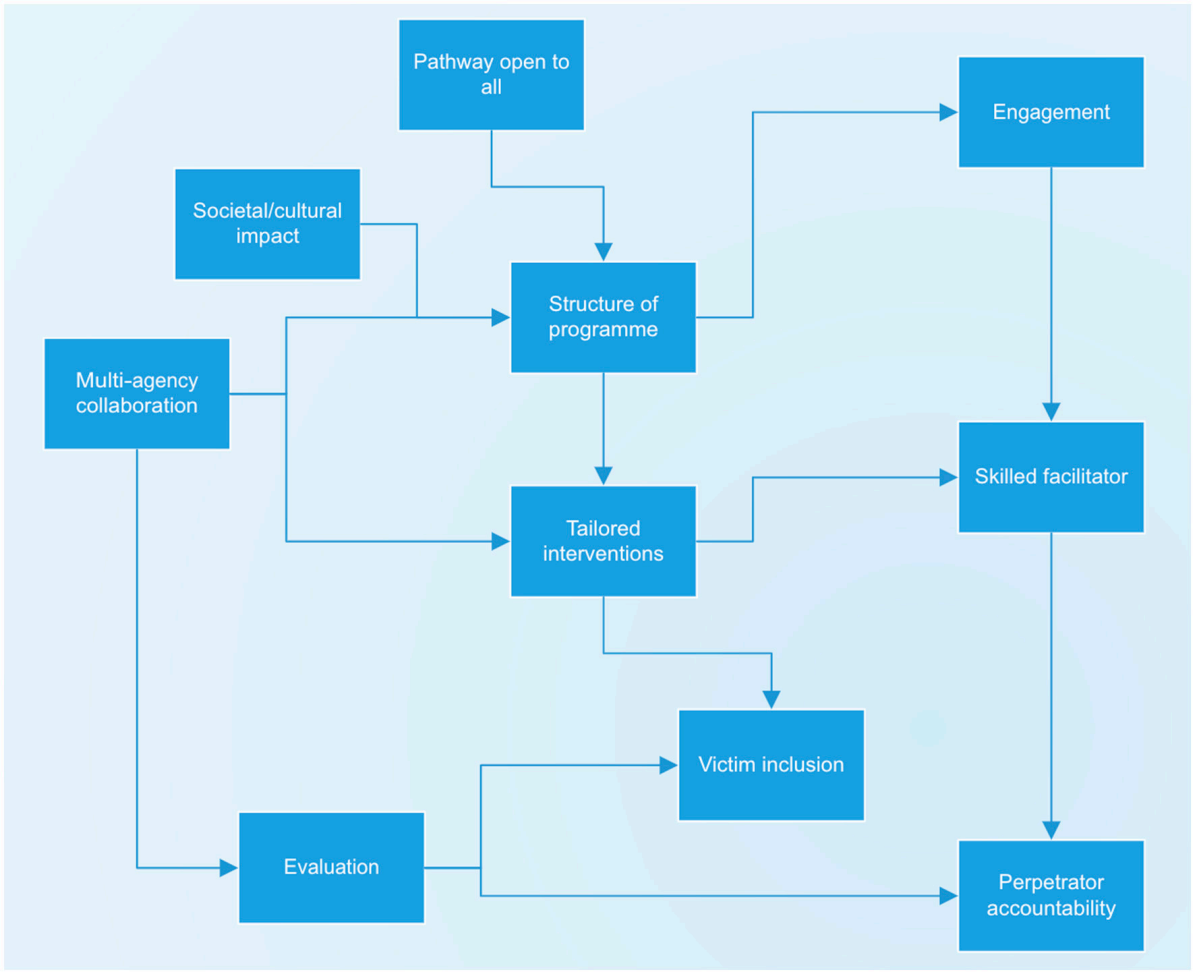
Overview of the themes' connections.

When considered together, the themes illustrate an interconnected framework that highlights how effective delivery programmes and outcomes can be achieved. For example, at the center of Figure 2 lies the structure of the programme, shaped by socio-cultural factors, multi-agency collaborations, and pathways of referral open to all participants. These foundational elements suggest that tailored interventions should be structured in ways that consistently meet individual needs and contexts. Active engagement with the programme and the presence of a skilled (or specialized) facilitator were also two crucial factors ensuring higher delivery quality. Collectively, the themes identified are believed to promote victim/survivor inclusion while strengthening the chances of accountability from perpetrators. From a less direct perspective, ongoing evaluation and progress underpinned and informed all aspects of the framework, ensuring that learning from practice and hindrances drives more effective improvement and contributes to more sustainable social-wide change.

## Discussion

The study identified ten key themes underscoring the essential role of perpetrator programmes in addressing DA. The findings reinforce previous research and emphasize that interventions must be tailored, evidence-based, and designed to address the diverse needs of those who cause harm, while consistently prioritizing the safety and wellbeing of victims and survivors (Home Office, 2023; Sabri et al., 2021; Safe Lives, 2023; Vall et al., 2024). When programmes prioritize accountability, challenging harmful beliefs and behaviors, and are facilitated by well-trained professionals, a greater potential for long-term impact is registered (Gondolf, 2012; McGinn et al., 2020). The structure and content of DA perpetrator programmes appeared, therefore, crucial to their success. Overall, content must be both accessible and theoretically sound, as well as practically relevant. Tools such as the “colors” session, derived from transactional analysis, and approaches informed by CBT were highlighted as particularly useful in the literature, and by clients of Up2U, when adequately adapted and applied (Pearson et al., 2022; Rathod et al., 2019). Facilitators and participants alike recognized the value of interventions that supported self-awareness and reflection, but concerns were raised about some content that was overly repetitive or abstract. To maintain engagement and facilitate change, programmes should avoid excessive cognitive load, regularly update materials, and reflect the evolving social and cultural contexts in which abuse occurs (Vall et al., 2024).

Informed by empirical insights across all study phases, a new 22-week Up2U programme structure was developed, encompassing four interconnected stages: assessment, foundations for change, behavior change, and sustaining change. This structure reflects a progression in participant insight and capacity for change. Tools such as the “Helicopter View” and “Time Out” techniques were seen as especially effective in promoting behavior regulation and helping individuals reflect on situations from different perspectives. When such tools are linked to real-life situations and internalized by participants, they can disrupt harmful patterns and reduce the likelihood of further abuse. In this new potential outline for a programme, the role of the facilitator is central to guaranteeing programme effectiveness. Staff delivering the programme must be equipped with a deep understanding of the theoretical underpinnings of the material, as well as the ability to adapt content responsively in the moment (Lila et al., 2018). The need to challenge participants, particularly in response to denial, minimization, or victim-blaming, must be balanced with respect and engagement. Some participants demonstrated the capacity to use self-reflection tools to justify or rationalize their violent behavior, making it imperative for facilitators to critically assess client responses and address manipulative dynamics when they arise (Gondolf, 2012; McGinn et al., 2020). Ongoing training, supervision, and opportunities for peer learning were identified as necessary supports for facilitators to deliver the programme effectively, maintain fidelity to its aims, and safeguard their wellbeing.

Multi-agency collaboration also emerged as a key factor in programme delivery. Referral pathways need to be informed by a thorough understanding of programme content to ensure that clients are appropriately matched and that agencies can offer consistent support. However, the delivery of such programmes is frequently hampered by inadequate resourcing (Lilley-Walker et al., 2018; Vall et al., 2024). There is a pressing need for targeted investment in skilled facilitation, one-to-one delivery capacity, and embedded victim support and engagement. While investment in this area may be politically challenging, the wider benefits (e.g., reduced harm, improved safety, and long-term economic savings) provide a strong case for prioritization (Gondolf and Jones, 2001; Turner et al., 2023). Victim/survivor involvement must therefore be embedded at all stages of intervention and evaluation. Their perspectives are crucial in assessing whether change has occurred and in ensuring that interventions do not increase risk. Yet, engaging survivors remains difficult, often due to safety concerns or emotional strain. Despite these challenges, failing to offer support is ethically untenable. Strengthening partnerships between perpetrator programmes and DA services, and involving people with lived experience in service design, may help improve engagement and responsiveness.

Measuring outcomes is also essential, but reliance solely on self-report or recidivism data presents documented limitations. Instead, a multi-level evaluation framework is recommended to evaluate Up2U's effectiveness, capturing participant engagement, behavioral learning, application in daily life, and broader social outcomes (Table 2). Tools such as Kirkpatrick's model (Kirkpatrick, 1998) offer a practical structure for assessing satisfaction, learning, behavioral transfer, and impact.

**Table 2:** Evaluation options and potential contribution to Up2U.

**Level**	**Kirkpatrick stages**	**Potential application for Up2U**
Level 1 reaction	Measured participant satisfaction with the programme and how useful they find it	•One-to-one feedback to facilitator •Measure engagement in activities •Post-session surveys •Post-programme surveys
Level 2 learning	Measured how much participants have learned in terms of skill, knowledge, attitude, and confidence, and engagement	•Pre and post-programme questionnaires •Feedback from the facilitator and other services that have connections to the participant •Stability throughout the programme
Level 3 behavior	Measured how much the participants implement what they have learned and apply it in practice	•Feedback from partner/ex-partner •Feedback from children •Repeat offending rates
Level 4 results	Measured the broader organizational outcomes and success measures, and, in this case, also societal impact	•Financial analysis: invisible cost savings •Reduced recidivism

Taken together, these findings point to the need for responsive programmes, theoretically informed, and survivor-centered. They must be adequately resourced, delivered by critically reflective practitioners, and continuously evaluated through diverse, robust measures. Perpetrator programmes, when effectively designed and implemented, offer a meaningful contribution to preventing abuse and fostering safer communities.

In summary, the recommendations from this study emphasize the need for perpetrator programmes to be evidence-based, regularly reviewed, and inclusive of feedback from facilitators, professionals, clients, and victim/survivors. Programmes should be accessible at the point of need and designed to strike a balance between respect and accountability, utilizing engaging and flexible content tailored to participants. Clear promotion and effective communication with referral agencies and clients are essential, including the use of preparation materials. Programmes must prioritize the safety of victims and children, offer holistic and sustained support beyond attendance alone, and be embedded within a multi-agency response. Facilitators require ongoing training, supervision, and support, and systematic evaluation should monitor behavioral change, engagement, cost-effectiveness, and victim/survivors' perspectives. Resourcing for these programmes must be aligned with investment in victim services to ensure meaningful impact.

## Limitations

While the study provides meaningful insights, its limitations must be acknowledged. The most significant constraint was the small number of direct participants with lived experience—only one victim/survivor and two individuals who had used harmful behaviors. Resistance to participation is widely attributed to factors such as stigma, fear of exposure, or emotional distress. In line with this, despite the implementation of clear ethical procedures and comprehensive participant information, recruitment remained a challenge. To moderate the effect of low participation, the secondary analysis of feedback questionnaires was undertaken, which helped to broaden the understanding of client experiences. Another limitation is the fact that the report did not address those under 18 who cause harm. While the authors argue that understanding teenage DA is crucial to improving legislation, its investigation fell outside the study's remit. Specialist responses for young people require separate consideration, particularly with regard to safeguarding, developmentally appropriate content and educational strategies focused on healthy relationships and emotional regulation, which could be explored in future research. A further limitation was the gender bias, although already noted that women are more likely to experience DA, this does not diminish the need for support for male victims and behavior change programmes for women who use harmful behaviors, especially as only around 4.8% of male DA victims request support in the UK (Office for National Statistics, 2024). Moreover, the authors also recognize that IPVA occurs in non-heteronormative relationships and that those in the LGBTQ+ community can also experience DA; it is just that these victims were not represented in the sample.

## Conclusion

The study highlighted the importance of flexible, evidence-based DA perpetrator programmes that prioritize victim safety while holding perpetrators accountable. Ten main themes were considered to provide timely and relevant recommendations for improving the current Up2U provision. The findings uniquely contribute to a multi-phase and multi-method evaluation of how to improve the programme engagement, provision, and long-term impact.

## Data Availability

The datasets presented in this article are not readily available. Requests to access the datasets should be directed to the corresponding author, harveyo@bournemouth.ac.uk.

## Footnotes

1 In the UK context, a high-harm offence is an umbrella term for crimes that are associated with substantial physical and psychological damage to victims. Some high-harm offences include, but are not limited to, murder, rape, grievous bodily harm (GBH) with intent, and non-fatal strangulation. The term “high-harm offence” is used operationally by criminal justice agencies and police constabularies to identify offenders posing higher risks of recidivism, and of causing future harm to survivors (College of Policing, 2021).

2 This category includes instances where, after the suspect was identified and reported to the police, the victim decided not to proceed with the allegations. This is a very common crime outcome that has been linked to fear of repercussion and secondary victimization. Most importantly, this outcome does not mean that the DA offence did not occur.

3 This is an overarching term for police outcome codes where either the police or the CPS decided not to proceed with the investigation despite the victim continuing to be supportive of the case, and might be due to external difficulties like the investigation not being in the public interest or the suspect not being identified.

4 The effect on recidivism refers specifically to how much an intervention or programme changes the likelihood that individuals will reoffend after release or participation. In contrast, the efficacy of programmes is a broader concept that examines how well a programme achieves its intended goals, which may include, but are not limited to, reducing recidivism. Programme efficacy can involve improvements in areas such as employment skills, attitudes, mental health, or social reintegration. In other words, while a programme's efficacy concerns how effectively it delivers its intended benefits, its effect on recidivism specifically measures whether those benefits translate into lower rates of reoffending. A programme may therefore be effective in achieving certain outcomes but still have a limited impact on recidivism, specifically if those outcomes do not directly address the factors that lead to reoffending.

5 The Up2U programme, developed by Portsmouth City Council and delivered in Dorset Council and Bournemouth, as well as Christchurch and Poole (BCP) Council, supports individuals aged 16 and over who use abusive behaviors in intimate relationships, including both heterosexual and same-sex partnerships. It offers personalized training aimed at promoting non-abusive behavior. Participation requires individuals to acknowledge their abusive actions and demonstrate a willingness to change. The programme also aims to provide support to current or former partners to ensure ongoing risk management. Its primary aims are to reduce incidents of abuse, break the cycle of harmful behavior, and decrease the number of children subject to child protection interventions.

6 Ketso boards are interactive, access-friendly toolkits designed to facilitate group discussion, reflection, and collective brainstorming. It consists of a reusable felt workspace and a set of color-coded leaves or shapes on which participants write their ideas, which are then placed on the board to visually organize and connect themes (Furlong and Tippett, 2013). The tool promotes inclusive participation, ensuring that all voices can contribute to the development of shared insights and solutions.

7 Both the interview schedule and the survey questions are available upon request.

8 This is technique that draws on the work of Allen (2001) referring to the mental process of systematically capturing all stray thoughts, and tasks, occupying your mind so that you can later organize and act on them.
